# Effects of Exercise Training on Autonomic Function in Chronic Heart Failure: Systematic Review

**DOI:** 10.1155/2015/591708

**Published:** 2015-10-12

**Authors:** Chung-Yin Hsu, Ping-Lun Hsieh, Shu-Fang Hsiao, Meng-Yueh Chien

**Affiliations:** ^1^Department of Physical Therapy and Rehabilitation, Zhongxing Branch of Taipei City Hospital, Taipei 10341, Taiwan; ^2^Department of Rehabilitation, Keelung Hospital, Ministry of Health and Welfare, Keelung 20147, Taiwan; ^3^School and Graduate Institute of Physical Therapy, College of Medicine, National Taiwan University, Taipei 10051, Taiwan; ^4^Department of Physical Therapy and Rehabilitation, National Taiwan University Hospital, Taipei 10002, Taiwan; ^5^Physical Therapy Center of National Taiwan University Hospital, Taipei 10002, Taiwan

## Abstract

*Objectives*. Cardiac autonomic imbalance accompanies the progression of chronic heart failure (CHF). It is unclear whether exercise training could modulate autonomic control in CHF. This study aimed to review systematically the effects of exercise training on heart rate recovery (HRR) and heart rate variability (HRV) in patients with CHF. *Methods*. Literatures were systematically searched in electronic databases and relevant references. Only published randomized controlled trials (RCTs) focusing on exercise training for CHF were eligible for inclusion. Outcome measurements included HRR and HRV parameters. *Results*. Eight RCTs were eligible for inclusion and provided data on 280 participants (186 men). The participants were 52–70 years of age with New York Heart Association functional class II-III of CHF. Each study examined either aerobic or resistance exercise. Two trials addressed outcome of HRR and six HRV among these studies. Two RCTs showed that moderate aerobic exercise could improve HRR at 2 minutes after exercise training in CHF. Five of six RCTs demonstrated positive effects of exercise training on HRV which revealed the increments in high frequency (HF) and decrements in LF (low frequency)/HF ratio after training. *Conclusion*. Participation in an exercise training program has positive effects on cardiac autonomic balance in patients with CHF.

## 1. Introduction

Chronic heart failure (CHF), the common final stage of all heart diseases with negative impact on prognosis, is increasingly prevalent worldwide and is associated with significant morbidity and mortality [1, 2]. Exercise intolerance is one of the major symptoms of CHF which can be contributed to several factors, including reduced cardiac output, cardiac cachexia, and neurohormonal axis changes [3, 4]. Autonomic imbalance includes increase in sympathetic tone, decrease in parasympathetic activity, and depressed heart rate variability (HRV) [5], which is a common clinical predictor of poor survival in CHF [6, 7]. Autonomic modulation is consequently an important issue of modern heart failure management.

The heart rate response to exercise and recovery from exercise depends on the dynamic interaction between the sympathetic and parasympathetic nervous systems [8]. Heart rate recovery (HRR) after exercise termination is mediated by a combination of sympathetic withdrawal and parasympathetic reactivation, primarily by vagal reactivation [9]. Slow HRR has been reported to be important in predicting mortality in healthy individuals [10] and people with heart failure [11]. HRR at 1 minute after exercise termination (HRR_1_) was thus used as a simple measure indicative of decreased autonomic nervous system activity [12]. HRV is another noninvasive and easy-to-obtain measurement of cardiac autonomic system function. HRV is defined as beat-to-beat variations in heart rate of individuals in sinus rhythm [13]. Reduced HRV generally indicates either failure or attenuation in the autonomic regulation of the sinoatrial node [13]. Although HRV and HRR do not directly measure autonomic nervous activity, both are considered significant prognostic indicators of mortality in CHF by evidence [14–16]. A variety of drugs as well as numerous invasive procedures have been reported to effectively modify HRV. However, evidence of its adverse events and sustained efficacy is lacking. In view of large number of other drugs that patients with CHF frequently take in conjunction with cardiac medication, an evidence-based nondrug approach is of interest.

Current evidence has recommended exercise training as a key component in the guidelines for secondary prevention of CHF [17]. Research in exercise training confirmed significant improvements of clinically relevant outcome parameters such as exercise capacity, quality of life, and CHF related hospitalization [18–20]. However, these studies were not designed to exclusively address outcome parameters such as HRV and HRR in patients with CHF. Aerobic exercise and physical training have been shown to improve HRV in various populations, such as athletes and sedentary individuals [21], and patients with cardiovascular diseases [22]. Despite the fact that exercise training is recommended as an adjunct to clinical therapy in patients with CHF [17], limited published data exist to evaluate HRV and HRR after exercise training in CHF. Because of easy access and a low cost, exercise training may be an alternative and favorable approach to existing therapies for prevention and treatment of autonomic imbalance among CHF populations. Therefore, our objective of this systematic review was to investigate the effects of exercise training on HRR and HRV in patients with CHF.

## 2. Methods

### 2.1. Identification and Selection of Trials

Five electronic databases (PubMed, the Cumulative Index to Nursing and Allied Health Literature [CINAHL], EMBASE, the Cochrane Library, and Chinese Electronic Periodical Service [Airiti Library]) from the earliest available date to March 2015 using various combinations of keywords for heart failure (*congestive heart failure, chronic heart failure*), for exercise (*exercise training, physical training*), and for autonomic (*autonomic function, heart rate recovery, heart rate variability*) were processed. We limited the search results to full-text articles in English or Chinese. We then checked the reference lists of the original and review articles that the initial search had yielded in order to identify additional full-text articles.

The inclusion criteria are presented as follows.

*Design*

- Randomized trial.

*Participants*

- Chronic heart failure.
- Ejection fraction < 40%.

*Intervention*

- Exercise training program (aerobic or resistance exercise).

*Outcome Measures*

- Heart rate recovery.
- Heart rate variability.

*Control*

- No training or usual care.

Randomized controlled trials (RCTs) eligible for subsequent criteria were included in this systematic review. Interventions were based exclusively on exercise training, aerobic or resistance training modules alone, or the combination of both. Trials were excluded if the group allocations are not pure control group of CHF patients versus an exercise group and if the other forms of physical therapy were applied.

Two reviewers (Hsieh and Hsu) independently reviewed the articles to determine whether the articles met the predetermined eligibility criteria. The results were rechecked by the senior authors (Hsiao and Chien), and all reviewers resolved any disagreement and ambiguous or equivocal information through discussion and writing letter of confirmation to the authors to reach a consensus in every relevant detail. In cases of multiple publications arising from a single trial, only the report that contained the most detailed, updated, and quantified information regarding both intervention and outcomes was included.

### 2.2. Assessment of Characteristics of Trials

*Quality*. The methodological quality of the selected trials was independently assessed by two reviewers (Hsieh and Hsu) using the Physiotherapy Evidence Database (PEDro) scale. Any disagreement with regard to methodological quality was resolved through discussion and consensus.

*Participants*. Demographic data such as age, gender, New York Heart Association (NYHA) functional class, and ejection fraction (EF) were recorded to characterize the trials and to determine the homogeneity of participants between groups and between trials.

*Intervention*. The target intensity, duration, frequency and the total period of time for exercise training program, and the nature of the control group were recorded.

*Outcome Measures*. The measured outcomes we considered were HRR at 1 or 2 minutes after exercise termination (HRR_1_, HRR_2_) and time domain of HRV (RR interval, standard deviation of all RR intervals [SDNN], root mean square of difference in RR intervals [RMSSD]), and percentage difference between adjacent NN intervals (pNN) and frequency domain of HRV (high frequency (HF), low frequency (LF), and LF/HF ratio).

## 3. Results

### 3.1. Flow of Studies through the Review

**Figure 1:**
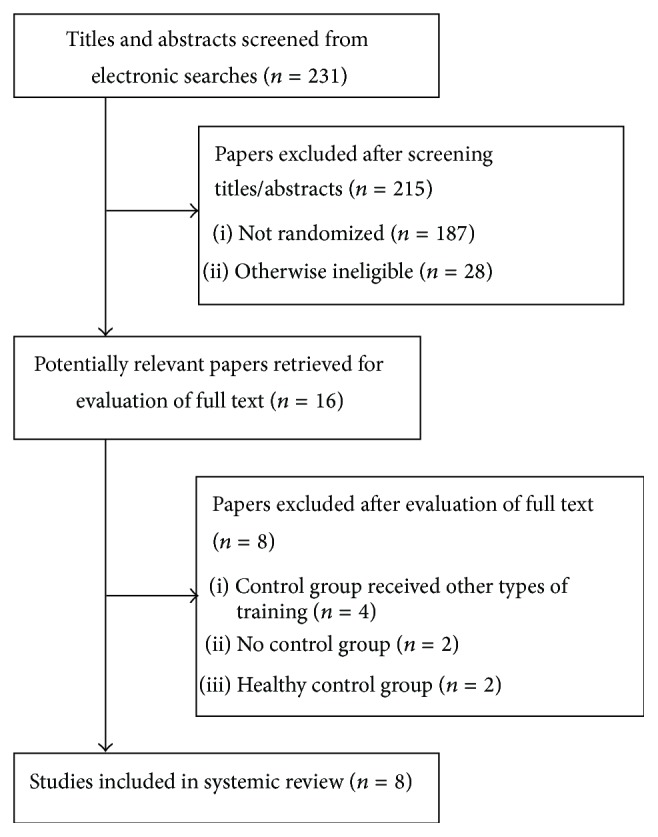
Trial flow diagram of this systemic review.

Initially, 231 studies have been identified through the database search of which 16 were considered potentially relevant and respected the previously mentioned inclusion criteria. Out of these, 8 eligible articles were retained for further systematic review after the screening of titles and abstracts [23–30]. Eight articles were subsequently excluded in which 4 trials had their control groups engaged in some forms of exercise [31–34], 2 trials had no control group [35, 36], and 2 trials had control groups of normal healthy participants that did not meet the inclusion criteria [37, 38] (Figure 1). No additional articles were identified by the scanning of reference lists. Therefore 8 trials were included in the analysis.

### 3.2. Characteristics of Included Trials

The methodological quality of selected trials assessed by the PEDro scale is shown in Table 1 and a summary of the trials is presented in Table 2.

*Quality*. Based on the quality of PEDro scale for methodological quality assessment, the RCTs included in this systematic review are of good quality with six trials scoring 7/10 [23, 24, 26–29] and two trials with fair scoring of 5/10 [25, 30]. No trial blinded participants or therapists, while all trials blinded assessors. Most trials had retention rates of 85% or greater and reported between-group differences with point estimates and measures of variability (Table 1).

*Participants*. The eight included trials involved 280 participants (186 men and 94 women) with sample sizes averaged from 20 to 66 for each study. The majority of the patients were 52–70 years of mean age with at least 6-month diagnosis and treatment of CHF. The subsets of the diagnosis included ischemic, coronary artery, hypertensive, valvular heart disease, and idiopathic dilated cardiomyopathy. Except for 3 trials [23, 27, 29], all the other trials contain patients with NYHA functional class II-III with their EF < 40%. Their comorbidity included hypertension, diabetes, coronary heart disease, prior myocardial infarction, and coronary bypass grafting. The pharmacological therapy included angiotensin-converting enzyme inhibitors, beta-blockers, statins, digoxin, and diuretics.

*Interventions*. Six trials examined supervised aerobic exercise (walking, cycling ergometer, or Tai Chi) [23, 24, 26–29] and two examined supervised resistance training (multistation hydraulic resistance training or circuit weight training) [25, 30]. The duration of the trials was between 8 and 24 weeks, most trials with 12 weeks. Exercise time for each session of the trials varied from 30 minutes to 1 hour. The frequency of exercise programs was between 2 and 5 times per week, most trials with 3 times per week. The control groups in all the trials received either no treatment or health education. Most aerobic exercise programs examined were of moderate intensity, instructing the participants to reach 50% to 80% of their heart rate reserve or peak oxygen consumption for 20 to 60 minutes (Table 2).

### 3.3. Effect of Exercise Training on HRR and HRV

#### 3.3.1. Trials with HRR as Outcome Measure for Exercise Training

Two trials investigated the effects of exercise training on HRR [23, 24]. Out of that, one trial evaluated both HRR_1_ and HRR_2_ [24], and the other utilized HRR_1–6_ as outcome measures [23]. These 2 trials employed 8- and 12-week moderate intensity aerobic exercise training program. The results of both trials showed no significant effect on HRR_1_; however, their HRR_2_ manifested significant improvement (10.9 and 24 bpm, resp., *P* < 0.05). The results of study by Myers et al. [23] even showed statistically significant improvement in HRR_2–6_ (*P* < 0.05). In summary, it was shown that moderate intensity aerobic exercise training improved HRR in patients with CHF.

#### 3.3.2. Trials with HRV as Outcome Measure for Exercise Training

Six RCTs investigated the effects of exercise training on HRV in which 3 trials employed short-term recordings of HRV [25–27] while 3 others employed 20- to 24-hour Holter electrocardiogram (ECG) recordings of HRV [28–30]. Four out of the 6 trials analyzed HRV parameters in both time and frequency domains. One trial utilized only time domain analysis [26] and one frequency domain [27]. Five trials (4 aerobic exercise program and one resistance exercise program) showed some improvements in time and frequency domain HRV parameters. A significant increase in both SDNN and RMSSD (15.46 and 17.56 ms, resp., *P* < 0.05) after exercise training was reported [26]. In addition, several studies revealed the increments in HF as well as the reduction in LF/HF ratio after exercise training in patients with CHF [25, 27, 28]. Only one trial which consisted of resistance training and HRV parameters obtained by 24-hour Holter did not find any significant change of HRV after exercise training [30]. In an overview of the results, moderate intensity aerobic exercise training was effective to ameliorate HRV in patients with CHF.

#### 3.3.3. Adverse Events

No adverse event relevant to exercise training during these trials occurred or was reported.

## 4. Discussion

This was the first systematic review providing a comprehensive survey of RCTs which examined the effects of exercise training on autonomic function in patients with CHF. Although the heterogeneity of HRV parameters restricted the direct pooled analysis, the results derived from fair to good quality evidence indicated that participation in exercise training programs which consisted of moderate intensity aerobic exercise had beneficial effects on autonomic function, as indicated by increases in HRR as well as HRV parameters. Since both slow HRR and attenuated HRV predict adverse health outcomes in patients with CHF, optimal exercise prescription should not only aim to improve exercise capacity but also focus on autonomic function in patients with CHF. Physical training could be considered as an alternative approach for autonomic dysfunction in patients with CHF.

The ability of heart rate to recover after exercise is related to the capacity of the cardiovascular system mediated by vagal activity and baroreceptor adaptations that occur during exercise [9]. HRR can be an additional indicator of outcome measures and risk stratification in patients undergoing cardiac rehabilitation [23]. This systematic review showed that moderate-intensity aerobic exercise training was effective in improving HRR, especially HRR_2_. It was reported that HRR_1_ is considered a marker of cardiac parasympathetic outflow, and HRR_2_ is thought to be related to the gradual withdrawal of sympathetic activity [39]. Both parameters are of considerable importance to cardiac patients. However, HRR_2_ was reported to be superior to all other time periods as a mortality predictor [40].

The magnitude of improvement of HRR seemed to be associated with the improvements in fitness levels of the patients [24]. Several studies have reported that changes in HRR were attributed to a greater heart rate reserve after training [23, 41] but do not negate the potential influence of training on autonomic balance. An increase in vagal tone after training is implied by the reduction in resting heart rate, and the higher peak heart rate suggests enhanced sympathetic drive, lowered vagal influence, or both at peak exertion during exercise. These all indicated exercise training provided a benefit to autonomic control.

Reduced HRV in patients with CHF is often thought to be related to neurohormonal activation and attenuation of cardiac vagal tone [5, 16]. Different HRV assessment contexts (e.g., short-term versus 24-hour recordings) seemed to have some influences on the results. The effect of exercise training was likely to be more prominent in the studies utilizing a short-term resting ECG recording in our included trials. The time domain HRV parameters reflect overall autonomic modulation with parasympathetic components well represented by the RMSSD and pNN50 parameter. In frequency domain HRV analysis, it is generally accepted that the HF is reflective of parasympathetic activity, while the LF reflects both sympathetic and parasympathetic activity and is now believed to represent baroreflex sensitivity instead of sympathetic modulation [42, 43]. Sympathovagal balance is frequently described by LF/HF ratio. Recently, LF/HF ratio represents a relationship between baroreflex sensitivity and vagal modulation rather than sympathovagal balance [44]. There is no HRV parameter reflecting directly sympathetic activation modulation. The results of this review revealed that exercise training has considerable effects on HRV in patients with CHF, including increase in vagal tone and modulation of sympathovagal balance activity.

Two out of the 8 RCTs employed resistance exercise and 6 trials employed moderate-intensity aerobic exercise training. Most of these results showed positive exercise training effect on the autonomic nervous system regulation in CHF. Only one trial did not show the effect of HRV after exercise consisting of resistance training in these trials [30]. Therefore, moderate intensity aerobic training is currently evidenced as the main adjunct in improving autonomous regulatory function in CHF. However, there were different studies addressing the effects of different intensities of aerobic exercise on autonomic function in CHF. Dimopoulos and colleagues [31] conducted a study to compare the effects of moderate intensity continuous exercise and high intensity interval exercise (100% peak work rate for 30 s and alternating with rest for 30 s) training on HRR_1_ in patients with CHF. This study was not eligible for inclusion because they did not include a real control group. There were 24 stable CHF patients who completed a rehabilitation program of 36 sessions, three times per week. The results showed that moderate intensity continuous exercise rather than high intensity interval exercise training improved early HRR_1_. Future studies should pay more attention to the issues about optimal exercise intensity. On the other hand, more studies are required to investigate the effects of resistance exercise training in this population.

In addition to exercise intensity, the training duration would be the other possible factors affecting the effects of exercise program for autonomic function. The mean follow-up duration in our included clinical trials was relatively short (ranging from 12 to 24 weeks). A longer follow-up duration after exercise training might amount to some contrasting effects on HRV and HRR since it has been suggested that CHF patients may require more time to achieve modulation of autonomic tone and responsiveness [24, 45].

Due to a great variability of evaluation tools and analytical methodology across trials, direct comparison and pooling of the data was restricted. We calculated the effect sizes of some of the included RCTs, but not all, because some trials did not report the detail data. Generally, the studies eligible for inclusion into our systematic review showed that exercise training improved autonomic function within moderate effect sizes ranging from 0.49 [24] to 0.70 [27]. More studies with larger effect sizes are needed to provide better evidence.

The mechanisms by which exercise improves autonomic function are not well understood. Some mediators are considered to play a role in increasing cardiac vagal tone in response to exercise training [46, 47]. Nitric oxide (NO) is thought to have an effect on cardiac vagal tone and sympathetic influence; on the other side, angiotensin II is a known inhibitor of cardiac vagal activity. Exercise training has been shown to improve NO bioavailability and lower angiotensin II levels [46, 47]. In addition, recent studies have shown that chronic inflammation affected the autonomic nervous system [48]. Interleukin-6 may affect autonomic balance by disturbing the hypothalamic-pituitary-adrenal axis at the level of the pituitary and adrenal glands [49]. The anti-inflammatory effects of exercise training might be a possible mechanism by which exercise improves autonomic function. Nevertheless, future studies are needed to explore this issue.

## 5. Limitations

The systematic review combined the results of different studies; nevertheless, several limitations in generalizing the findings must be acknowledged. First, a relatively small number of trials, all of which included a relatively small sample size, were examined. Second, trials reported in languages other than English and Chinese were excluded, as were trials reported only as abstracts. These exclusions may have led to publication bias. Third, considerable variations in the parameters analyzed and the HRV assessment contexts (e.g., short-term versus long-term recordings of HRV) all restricted direct comparisons of the data and pooled analysis. Finally, the exercise modes, intensity, and duration varied among trials that hindered determination of the optimal exercise prescription parameters on HRR and HRV.

## 6. Implications and Recommendations for Future Study

According to the results of this systematic review, moderate-intensity aerobic training utilizing walking or bicycle ergometer in 50–80% of heart rate reserve, 30 minutes to 1 hour, 3 to 5 times per week for at least 12 weeks may be recommended for amelioration of the autonomic regulation in people with CHF. Further research could examine additional aspects of the effects of exercise training in this population, for example, the impact on the responses to exercise training under different levels of severity and the underlying causes of CHF. A threshold intensity or amount of exercise may be needed to affect cardiac autonomic function. The type of the exercise, such as high-intensity interval aerobic training or resistance training, may also influence the obtained effects.

## 7. Conclusion

HRR and HRV analysis provide noninvasive indicators to reveal the changes in the autonomic nervous system at rest and in response to physical activity in patients with CHF. This systematic review indicated that participation in exercise training has beneficial effects on ameliorating autonomic dysfunction in people with CHF. Large-scale, well-controlled, and longitudinal studies are needed to provide more evidence and further examine mechanisms that underlie the links between the effects of exercise training and autonomic function in CHF.

## Figures and Tables

**Table 1:** PEDro scores for included trials (*n* = 8).

Trials	Random allocation	Concealed allocation	Groups similar at baseline	Participant blinding	Therapist blinding	Assessor blinding	<15% dropouts	Intention-to-treat analysis	Between-group difference reported	Point estimate and variability reported	Total (0 to 10)
Myers et al., 2007 [23]	Y	N	Y	N	N	Y	Y	Y	Y	Y	7/10
Yaylali et al., 2015 [24]	Y	N	Y	N	N	Y	Y	Y	Y	Y	7/10
Selig et al., 2004 [25]	Y	N	Y	N	N	Y	N	N	Y	Y	5/10
Murad et al., 2012 [26]	Y	N	Y	N	N	Y	Y	Y	Y	Y	7/10
Ricca-Mallada et al., 2012 [27]	Y	N	Y	N	N	Y	Y	Y	Y	Y	7/10
Kiilavuori et al., 1995 [28]	Y	N	Y	N	N	Y	Y	Y	Y	Y	7/10
Yeh et al., 2008 [29]	Y	N	Y	N	N	Y	Y	Y	Y	Y	7/10
Cider et al., 1997 [30]	Y	N	N	N	N	Y	Y	Y	Y	N	5/10

N = no; Y = yes.

**Table 2:** Overview of included randomized control trials (*n* = 8).

Study	Participants	Intervention	Outcome measures	Main results
Myers et al., 2007 [23]	CHF (EF < 40%) Ex: *n* = 12 men, aged 56 ± 5 years Con: *n* = 12 men, aged 55 ± 7 years	Exercise: supervised hospital-based exercise training 1-hour walking and 45 min cycling, 5x/wk for 8 weeks at 60%–80% of heart rate reserve Control: usual care	HRR_1–6_	Exercise group got significant improvements in HRR_2–6_

Yaylali et al., 2015 [24]	CHF (EF < 45%, NYHA II-III) Interval: *n* = 13 men, 4 women, aged 63.7 ± 8.8 years Continuous: *n* = 13 men, aged 59.6 ± 6.8 years Con: *n* = 9 men, 2 women, aged 60.6 ± 9.9 years	Exercise 1 (interval training): 30 minutes of cycle ergometer aerobic exercise 3x/wk for 12 weeks at 50%–75% HRR Exercise 2 (continuous training): exercise protocol similar to interval training group without resting intervals Control: continued with ADL	HRR_1_, HRR_2_	Interval training group got significant improvements in HRR_2_. Unchanged HRR_1_ irrespective of any groups

Selig et al., 2004 [25]	CHF (EF < 40%, NYHA II-III) Ex: *n* = 15 men, 4 women, aged 65 ± 13 years Con: *n* = 18 men, 2 women, aged 64 ± 9 years	Exercise: supervised hospital-based 1-hour multistation hydraulic moderate intensity (according to heart rate monitoring) resistance training, 3x/wk for 3 months Control: usual care	Short-run rest ECG HRV: RR interval, SDNN, RMSSD, LF_nu_, HF_nu_, and LF/HF	Exercise group got significant decreases in LF, HF, and LF/HF after training

Murad et al., 2012 [26]	CHF (EF < 40%, NYHA II-III) Ex: *n* = 11 men, 20 women, aged 68.0 ± 4.8 years Con: *n* = 13 men, 22 women, aged 70.1 ± 5.6 years	Exercise: supervised hospital-based 1-hour walking and 15–20 minutes of cycling exercise training, 3x/wk for 16 weeks at 40%–50% to 60%–70% heart rate reserve Control: monitored with phone calls every 2 weeks	Short-run rest ECG HRV: SDNN, RMSSD	Exercise group showed significant increases in SDNN and RMSSD compared to controls

Ricca-Mallada et al., 2012 [27]	CHF (EF ≦ 40%, NYHA I-II) Ex: *n* = 8 men, 2 women, aged 59.0 ± 7.9 years Con: *n* = 8 men, 2 women, aged 56.5 ± 8.4 years	Exercise: supervised hospital-based 55-minute circuit bicycle resistance training, 3x/wk for 24 weeks at 50%–80% of HR reserve Control: usual medication	Short-run rest ECG HRV: RR interval, SDNN, LF, HF, and LF/HF	Exercise group got significant increases of mean RR interval, HF, and LF after training

Kilavuori et al., 1995 [28]	CHF (EF < 40%, NYHA II-III) Ex: *n* = 8 men, aged 52 ± 8 years Con: *n* = 11 men, 1 woman, aged 52 ± 10 years	Exercise: supervised hospital-based ergometer cycling for 30 minutes, 3x/wk for 3 months at 50%–60% of VO_2max_ Control: no changing normal ADL	20 h Holter HRV: HF, LF, VLF, LF/HF, and VLF/HF	Exercise group got significant changes in HF, VLF/HF, and LF/HF during the day

Yeh et al., 2008 [29]	CHF (EF ≦ 40%, NYHA I–IV) Ex: *n* = 10 men, 5 women, aged 66 ± 12 years Con: *n* = 9 men, 6 women, aged 61 ± 14 years	Exercise: supervised hospital-based Tai Chi training (1 hour), 2x/wk for 12 weeks Control: usual care	24-hour Holter HRV: SDNN, RMSSD, pNN10–50, LF, HF, and LF/HF	Exercise group showed trends towards increased pNN10–50 values during sleep, but not in the control group

Cider et al., 1997 [30]	CHF (NYHA II-III) Ex: *n* = 9 men, 12 women, aged 61.8 ± 9.8 years Con: *n* = 7 men, 12 women, aged 64.7 ± 5.3 years	Exercise: supervised hospital-based 60-minute circuit weight training at 60% 1-RM for 2 sets, 2x/week for 20 weeks Control: usual care	24-hour Holter HRV: time/frequency domain parameters	No significant difference between the two groups in all HRV parameters

ADL: activities of daily living; CHF: chronic heart failure; ECG: electrocardiogram; EF: ejection fraction; HRR: heart rate recovery; HRV: heart rate variability; NYHA: New York Heart Association; pNN10–50: percentage difference between adjacent NN intervals that are greater than 10–50 ms; SDNN: standard deviation of all RR intervals; RMSSD: root mean square of difference in RR intervals; LF: low frequency; HF: high frequency; VLF: very-low frequency; VO_2max_: maximal oxygen consumption.
